# Assertive community treatment for adults with complex mental illness and emergency department use: a systematic review

**DOI:** 10.3389/fpsyt.2026.1837300

**Published:** 2026-08-13

**Authors:** Vanessa von Berg, Hazel Heng, Md Shapin Ibne Sayeed

**Affiliations:** 1 1Mental Health Division, Northern Health, Epping, VT, Australia; 2 Department of Allied Health, La Trobe University, Bundoora, VT, Australia; 3 Allied Health Division, Northern Health, Epping, VT, Australia; 4 Public Health Department, Melbourne, VT, Australia

**Keywords:** assertive community treatment, complex needs, emergency department, mental health, psychiatry, psychosocial

## Abstract

**Introduction:**

Emergency Department (ED) wait times have increased globally, resulting in treatment delays and system strain. Complex mental health consumers, those with Serious and Persistent Mental Illness (SPMI), are a consistent subgroup of ED presenters. Assertive Community Treatment (ACT) has shown promise in reducing inpatient demand but has been criticized as cost-intensive with limited rehabilitation outcomes.  This systematic review evaluated ACT’s effectiveness in reducing ED usage and improving psychosocial outcomes.

**Methods:**

Nine databases were searched (31/12/2013–25/10/2023), yielding studies with moderate Risk of Bias scores. Studies spanned urban and rural areas across diverse geographic regions (Asia Pacific, Europe, North America).

**Results:**

ACT was associated with reductions in ED presentations and costs in seven studies. Improvements in psychosocial functioning were documented in five studies, although symptom reduction was limited. These findings were primarily based on clinician-reported Routine Outcome Measures (ROMs), with minimal inclusion of patient-reported outcomes, limiting interpretive reliability.

**Discussion:**

The evidence suggests that ACT programs reduce the incidence of, and costs associated with ED use and improve patient wellbeing (particularly social functioning) in participants with SPMI. The absence of comparisons between ED savings and ACT implementation costs restricts conclusions about sustainability. These results are limited in generalizability due to a moderate risk of bias related to confounding variables. Future research should explore client perspectives and cost-benefit analyses. In the interim, ACT may be an appropriate intervention for clients with SMPI and high ED usage who are unable to be managed via case management teams.

**Systematic Review Registration:**

https://www.crd.york.ac.uk/PROSPERO/view/CRD42023474465, identifier CRD42023474465.

## Introduction

Emergency department (ED) wait times are increasing, leading to treatment delays and increased mortality (1, 2). The Australian Institute of Health and Welfare (AIHW) 2023 report states that ED wait times have increased 5% nationally. Australian data indicate that approximately 90% of mental health (MH) attendees will spend more than 11 h waiting in the ED, a stark increase from the average wait of around 2 h (3). Similar patterns have been noted globally, with Denmark, Canada, and the USA reporting increased presentations and longer wait times for psychiatric patients (4–6).

A cohort within the population of presenting psychiatric patients comprises individuals with serious and persistent mental illness (SPMI). Consumers within the SPMI category experience chronic, often unremitting symptomology, complex health and social issues, and substance misuse, resulting in higher emergency service utilisation (1, 7, 8). This subset of patients is usually supported through a case management framework that aims to address biopsychosocial needs outside a crisis setting (9). A key feature of case management is that it is provided during business hours (Monday–Friday), which has previously been described as a limitation in servicing the SPMI cohort (9).

Despite community support, clients with SPMI continue to utilise EDs at high rates, citing unmet psychosocial needs (6). High ED presentation rates are problematic on multiple fronts. The resulting increase in wait times causes treatment delays for all patients and stretches hospital budgets (3, 9, 10). In MH consumers, prolonged ED wait times are associated with negative emotional states, distress and trauma (9, 11). Managing these patients ethically, safely, and in a manner that improves patient flow has become a key priority for health organisations (12).

Assertive Community Treatment (ACT), born out of deinstitutionalisation, is an intervention that provides intensive support via high-contact, *in vivo* practice across extended service hours (13, 14). Early studies showed that ACT is effective in reducing psychiatric admissions amongst patients with SPMI whilst improving psychosocial outcomes (13–16). However, from its height of popularity in the 1970s, ACT use has declined due to poor replicability, perceived intrusiveness, lack of client voice, and concerns regarding high implementation costs (14, 17). Consequently, ACT programmes have lost popularity in favour of less costly, existing case management models that offer less intensive support (9). Despite criticisms and waning use, ACT has continued to generate evidence of its ability to reduce hospital admissions (9, 15, 18). Hospitals and community organisations are turning to redesigned ACT programmes, which have demonstrated some utility in reducing crisis service presentations (18). This review explores the evidence regarding changes in ED use and treatment outcomes amongst ACT consumers to inform the development of new models of care.

This paper aims to conduct a systematic review of ACT’s efficacy in reducing ED use to assess its viability for implementation in Area Mental Health Services in metropolitan Australia. A secondary aim is to investigate psychosocial outcomes in this cohort, where reported. This review was undertaken in accordance with the Preferred Reporting Items for Systematic Reviews and Meta-Analyses (PRISMA) guidelines and was prospectively registered with the International Prospective Register of Systematic Reviews (19) (CRD42023474465).

## Methods

### Search strategy

Nine databases (scientific and grey), including CINAHL, MEDLINE, Scholar, Scopus, PsycINFO, PubMed, Social Care Online, Cochrane, and Social Work Abstracts, were searched between 31 December 2013 and 25 October 2023 to coincide with ACT’s re-emergence in community psychiatry. Keywords included “Assertive Community Treatment”, “mental health outreach”, “assertive community outreach”, “Flexible Assertive Community Treatment”, “adult”, “young adult”, “middle age”, and “emergenc*” (see **Appendix A** for the full search strategy). A hand search of the reference lists of included studies was completed. Duplicates were removed using bibliographic software (EndNote 20) and Covidence, a web-based collaboration software platform that streamlines the production of systematic and other literature reviews (20, 21).

### Eligibility criteria

Even though ACT is a clearly delineated model, variations of ACT have been developed over time to address criticisms or overcome service limitations (14, 22, 23). To ensure this study captured the breadth of current research, articles were included if they evaluated a high-contact, outreach-based mental health intervention by a multidisciplinary team. Additional inclusion criteria required that participants were adults (“adult”, “young adult”, “middle age”, indicating individuals over 18 years of age) and demonstrated behaviours and symptoms congruent with SPMI. Studies of paediatric cohorts were excluded in keeping with the service inclusion criteria. Studies were also excluded if a measure of ED use was not included. Studies were included regardless of whether psychosocial rehabilitation outcomes were reported to better understand the application of ACT in contemporary practice.

Title and abstract screening were conducted by two independent reviewers (VB, social work; BB, nursing). Both reviewers are senior mental health clinicians with previous experience of assertive outreach and complex care but are not currently employed in ACT teams. The full text of identified papers was retrieved and assessed by the same reviewers. Any conflicts were discussed and resolved (Kappa score = 0.52, indicating moderate agreement) before finalising the articles for inclusion in the study. A range of study designs was included for extraction to reflect the complexity of mental health research and provide understanding of the context, mechanisms of change and stakeholder experience of participants.

### Outcomes and data extraction

The primary outcome examined in this study was whether there was a change in ED use amongst consumers engaged in ACT. A secondary outcome examined changes in psychosocial functioning. Measures of client functioning in AMHS are most often obtained using scales that assess functioning across multiple domains over time (24). This systematic review synthesised these outcomes to address the study objective. Full-text articles were analysed using NVivo 14, where data were coded according to participant demographics, design features, intervention, comparison, and outcomes (25). Participant demographic data, changes in ED use, and psychosocial outcome measure scores were extracted and reported using basic descriptive statistics (mean, SD), whereas qualitative data were synthesised narratively.

### Risk of bias assessment

Included articles were assessed for risk of bias (ROB) using the Risk of Bias in Nonrandomised Studies (ROBINS-I) tool (26). The tool estimates internal validity between two or more interventions, resulting in a judgement of low, moderate, serious, or critical ROB (26). Eight studies were eligible for assessment using the tool and received ratings of low/moderate ROB. Two studies were ineligible for ROBINS-I assessment because they did not meet the criteria for testing (a report and a case study). The first was a peer-reviewed, government-commissioned report. The second was a case study that also lacked sufficient detail regarding its intervention.

## Results

**Figure 1:**
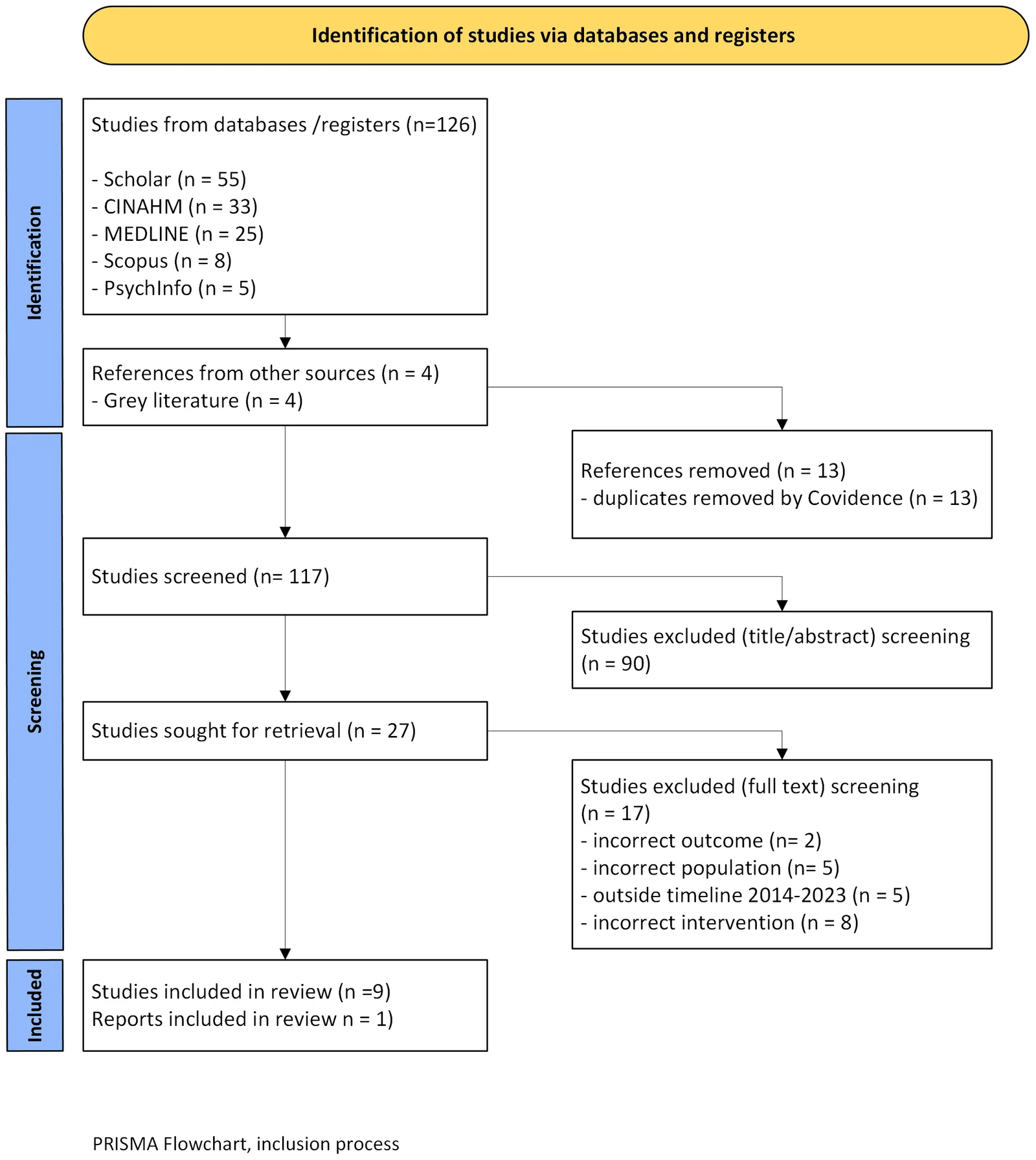
PRISMA flow chart of the inclusion process.

The primary search identified 130 studies from nine databases and grey literature. A total of 13 duplicates were removed, and 90 articles were excluded following title and abstract screening. A final 27 articles remained for full-text review, resulting in 10 articles being included for data extraction (**Figure 1**).

### Characteristics of studies

Included articles comprised seven quasi-experimental studies, one qualitative case study, one systematic review, and one report (**Table 1**). The systematic review reported on 10 healthcare−related ACT articles, including one that was embedded within another article identified during screening and subsequently included (27). Articles were sourced from countries in the Asia-Pacific, European, and North American regions. Among the included studies, ACT was most often delivered in urban settings. However, Kakuma et al. (28) also referenced programmes available in regional and rural areas of Australia, and Pope and Harris (29) conducted their study in a rural Canadian setting. The gender distribution of participants was relatively even, with a slight predominance of men (57%). This is supported by the research literature, in which the incidence and severity of affective psychosis presentations are higher in men (30, 31). Reported mean ages in the included studies ranged from 26.9 to 43 years, indicating a predominantly middle-aged population. Participants were most commonly diagnosed with psychotic illness and/or substance use disorder using the Diagnostic and Statistical Manual, Fifth Edition (DSM 5) (32) or the International Classification of Diseases, 11th Revision (33). However, a myriad of other mental illnesses meeting the criteria for SPMI were also reported.

**Table 1:** Descriptive characteristics of included ACT studies.

Study	Year	Design	Country	Location	*N*	Participant age (years)	Participant gender (%)	Diagnosis
Aagaard et al.	2016	Quasi-experimental	Denmark	Urban	240	M = 41 (20–80)	124 (52%) men; 116 (48%) women	ICD-10 Code + AOD, forensic, or ++admission
Aagaard et al.	2017	Quasi-experimental	Denmark	Urban	240	M= 41 (20–80)	124 (52%) men; 116 (48%) women	ICD-10 Code + AOD, forensic, or ++admission
Hastrup et al.	2015	Quasi-experimental	Denmark	Regional	174	Not reported	Not reported	ICD-10 F20, F22, F25, F31 + AOD, forensic, or ++admission
Kakuma et al.	2017	Multi-intervention report	Australia	Global	N/A	N/A	N/A	Any mental illness
Lee et al.	2015	Quasi-experimental	Hong Kong	Urban	210	M = 40 (18–65)	117 (56%) men; 93 (55%) women	Psychotic disorders
Morandi et al.	2017	Quasi-experimental	Switzerland	Urban	30	M = 38.9 (18–65)	22 (73%) men; 8 (26%) women	Substance use disorders
Nikdel et al.	2022	Case study	USA	Urban	1	29	1 (100%) man	Psychotic disorders, MDD, ADHD, Poly-SUD
Nordentoft et al.	2015	Quasi-experimental	Denmark	Urban	547	M = 26.6 (18–45)	322 (58.9%) men; 225 (41%) women	Psychotic disorders
Pope et al.	2014	Quasi-experimental	Canada	Rural	29	M = 43 (20–66)	24 (82%) men; 5 (17%) women	Substance use disorders
Vanderlip et al.	2017	Systematic review	USA	Global	N/A	N/A	N/A	Any mental illness + complexity

### Types of ACT

Included articles predominantly utilised established ACT models, but two studies described variants of ACT that still met the inclusion criteria (34, 35). The variants were described as assertive outreach mental health programmes with a single-clinician caseload and services limited to business hours. The duration of the interventions ranged from 1 to 4 years and was consistent with ACT intensive service delivery models.

### Routine outcome measures

Six of the 10 studies included results of measures of client functioning (27–29, 34–36), with various routine outcome measures (ROMs) utilised. The most common measures included the Health of the Nation Outcome Survey (HoNOS), Quality of Life index (QoL), and the Global Assessment of Functioning (GAF). Two studies included a patient-reported outcome measure (PROM) of psychosocial functioning: the Hong Kong Chinese Version World Health Organisation Quality of Life Measure, abbreviated version (WHOQOL-BREF-HK), and the Community Mental Health Evaluation Initiative (CMHEI) (27, 29). The remainder used clinician-reported scales.

### Primary outcome: ED use

ED use was predominantly measured by frequency. Four studies (29, 34, 37, 38) reported a statistically significant reduction in the frequency of ED presentations amongst ACT consumers compared with treatment as usual or a time-limited case management model (**Table 2**). An additional study (35) found a nonstatistically significant 3% decrease in ED presentations amongst ACT consumers. The included systematic review (36) reported “a general pattern towards reduced emergency room utilisation”. Qualitative evidence included a broad Australian report on outpatient mental health services, which indicated that ACT programmes were associated with reduced ED presentations (28). One study reported no difference between ACT and treatment as usual in reducing ED presentations (27).

**Table 2:** Outcomes by ACT study (frequency/cost of ED presentation and psychosocial rehabilitation scores).

Study	Intervention (length)	Comparison	Outcomes	
			Change in ED presentation/cost (statistical power)	Psychosocial rehabilitation scores (outcome tool)
Aagaard et al. (38); Denmark	ACT (2 years + 2 years f/up)	Treatment as usual (not defined)	ED frequency reduction (p < 0.05)a	Not reported
Aagaard et al. (37); Denmark	ACT (2 years)	Treatment as usual (not defined)	ED frequency reduction (p < 0.00)a	Not reported
Hastrup (39); Denmark	ACT (4 years)	Treatment as usual (not defined)	ED cost reduction (p < 0.065)	Not reported
Kakuma et al. (28); Australia	Multi-intervention report	N/A	ED frequency reduction (“…compared with standard care, resulting in fewer hospital admissions, emergency department visits”)	“Improvements in symptom severity, symptom frequency, and functioning compared with standard care” (multiple measures collated)
Lee et al. (27); Hong Kong	ACT (18 months)	Treatment as usual (not defined)	No change (p ≥ 0.05)	BPRS × time/group (*p* < 0.001; Brief Psychiatric Rating Scale) Social relationships/groups (*p* < 0.01; Quality of Life WHOQOL-BREF-HK)
Morandi et al. (34); Switzerland	ICMA (12 months)	ACT +critical time intervention	ED frequency reduction (p = 0.046)	Nonaccidental self-injury (*p* = 0.041); problem drinking or drug taking (*p* < 0.001); cognitive problems (*p* = 0.009); depressed mood (*p* < 0.001); problems with relationships (*p* < 0.001); problems with living conditions (*p* = 0.003); problems with occupation and activities (*p* < 0.001); appointment attendance (*p* = 0.001); psychotropic medication compliance (*p* = 0.003) (Health of the Nation Outcomes Survey)
Nikdel et al. (40); USA	ACT + assertive outreach (3 years)	Nil	ED cost reduction (“…ACT can substantially reduce the costs of hospital care whilst improving the outcome and patient satisfaction”)	Not reported
Nordentoft et al. (35); Denmark	OPUS (modified ACT; 2 years)	Treatment as usual (not defined)	ED frequency reduction (p = 0.11)	Symptoms: p = 0.08; functioning: p = 0.03; user satisfaction scale: p = 0.001 (Global Assessment of Functioning)
Pope et al. (29); Canada	ACT (30 months)	No comparator	ED frequency reduction (*p* < 0.05)a	Client satisfaction: 3.4 (SD = 0.6) (user-created scale)
Vanderlip et al.	Systematic review	N/A	ED frequency reduction (“general pattern toward reduced emergency room utilisation”)	“Three of five studies found improvement in physical functioning and satisfaction with treatment”

^a^ Wilcoxon signed-rank test.

A novel outcome relating to costs was reported in two studies (39, 40). Hastrup and Aagaard (39) examined costs and found a nonsignificant reduction in psychiatric ED costs (*p* < 0.60). A case study by Nikdel et al. (40) reported “substantial” reductions in hospital costs amongst participants treated with ACT.

### Secondary outcome: psychosocial outcomes

Five quantitative studies reported psychosocial outcomes focusing on functioning, social skills, symptom severity, and satisfaction (27, 29, 34–36). Significant improvements in functioning were reported in three studies (34–36), whereas significant improvements in social relationships were reported (27, 34) in two studies. Evidence of changes in symptom severity was more equivocal and was assessed only in four studies (28, 34–36). All studies reporting psychosocial outcomes relied on secondary sources (clinician-reported rather than patient-reported outcome measures), except in Pope and Harris (29), who found 90% of participants reported high satisfaction with their enrolment in ACT. This systematic review also noted that improvements in patient outcomes were observed even when a study was not successful in reducing ED use (27). This finding indicates that clients experienced positive outcomes from participation in ACT programmes despite no reduction in ED use.

## Discussion

This review explored ACT’s efficacy in reducing ED use amongst clients with SPMI and its potential to improve psychosocial functioning. The findings indicated that ACT reduced the frequency of ED use amongst consumers with SPMI (28, 29, 34–38). In addition, ACT was shown to reduce costs associated with ED use (39, 40). Results relating to psychosocial improvements were less clear-cut. Improvements were recorded in social functioning and relationships rather than symptomology, with this information obtained exclusively through clinician reports.

This systematic review indicates that ACT participants had reduced ED use and improved measures of social interaction. Relationships were a significant predictor of recovery in a study of clients with a psychotic illness (41). With this in mind, ACT’s high-intensity, extended-hour service provision may support reduced ED use amongst clients with SPMI because of the increased opportunity to engage with community care providers.

This review also found that two studies reported reduced hospital costs amongst consumers engaged in an ACT programme (39, 40). ACT would likely provide an attractive expedited discharge pathway for hospitals seeking to address bed demand whilst ensuring timely assertive care for a client with SPMI (5, 7). It is unclear, however, whether these savings are maintained when accounting for the higher implementation costs of ACT programmes relative to case management.

A key criticism of ACT in previous iterations has been the high programme costs associated with extended hours and higher staffing levels (14). Calculating the average cost of MH care (both ED and outpatient) is complicated by comorbidities and varying levels of complexity (42). The average cost of an ED presentation in Australia was reported as $891 in 2021–2022, whereas the average cost per community-managed phase of care was $2,781 (43). Historically, ACT and CM models have not differed significantly in terms of implementation costs, with more recent studies indicating ACT’s cost-effectiveness (44, 45). The articles included in this review did not compare costs between ED and outpatient episodes of care, ultimately limiting the ability to determine whether reductions in bed−based utilisation remain cost−effective once increased community care costs associated with ACT are considered. A comprehensive cost analysis comparing these models is therefore essential to establish the economic value of ACT alongside its benefits for consumers.

Pivoting to client factors, the current review indicated increased psychosocial function amongst ACT participants as measured by ROMS. Traditionally, a key component of measuring patient care, ROMS is a clinician-reported assessment of client functioning (46). However, current research advocates that client voice is necessary to build strong ethical interventions in complex MH care (28). PROMs are an underutilised method that incorporates clients’ self-rated experience and is advocated as essential for informing treatment programmes and policies (46, 47). However, despite criticisms regarding the lack of client voice in previous ACT programmes, only one article in this review included client-reported psychosocial data (29). It is therefore essential that future investigations into ACT incorporate the client perspective.

On review, all studies included were vulnerable to bias because of confounding variables, including cointerventions (medications, additional psychosocial interventions), and there was no apparent evidence of controls used to account for these effects. This limitation likely produced a low/moderate positive skew in the intervention effect. Furthermore, across all articles, interventions were described in limited detail, making it difficult to ascertain the degree of variation from validated ACT programmes. Confounding variables were not identified, and no information was provided by the authors about the controls used to counteract them, which may have had an unknown impact on the findings (48). Accounting for extraneous variables in MH research is problematic but not insurmountable (49). Future investigations into the utility of ACT in reducing ED use should address this area. Further limitations within the scope of the review include the inclusion of English-language and adult-only cohort studies, both of which limit generalisability.

Understanding that community mental health interventions are diverse and multifaceted, this systematic review included heterogeneous study designs. Attention was paid to ensuring that study models incorporating the core characteristics of ACT (e.g., *in vivo*, assertive, and complex presentation) were included. These diverse inclusions are a strength, as they reveal consistent patterns of evidence across a variety of contexts. The included studies capture the complexity of community mental healthcare and support policy and service design decisions by identifying real-world commonalities.

The findings of this review indicate positive outcomes associated with the use of ACT. To increase the applicability of this research, future investigation is needed to understand the potential savings associated with using ACT to reduce ED use and to evaluate the inclusion of PROMs. Doing so will strengthen decision-making when incorporating ACT into new models of care. Pending this future research, it would be reasonable to provisionally recommend ACT as an intervention for clients with SPMI and high ED use who are unable to be managed by case management teams.

## Conclusion

Overall, the evidence suggests that ACT programmes reduce the incidence of, and costs associated with, ED use and improve patient wellbeing (particularly social functioning) amongst participants with SPMI. The absence of comparisons between ED savings and ACT implementation costs restricts conclusions regarding sustainability. These results have limited generalisability because of a moderate risk of bias related to confounding variables. Future research should explore client perspectives and cost–benefit analyses. In the interim, ACT may be an appropriate intervention for clients with SPMI and high ED use who are unable to be managed by case management teams.

## Data Availability

The original contributions presented in the study are included in the article/**Supplementary Material**. Further inquiries can be directed to the corresponding author.
