# Supplementary material for: Assertive community treatment for adults with complex mental illness and emergency department use: a systematic review
Source: Front Psychiatry. 2026 Aug 13;17:1837300. doi: 10.3389/fpsyt.2026.1837300 (PMC13518556; doi:10.3389/fpsyt.2026.1837300)
Supplement: Supplementary file 1 [file DataSheet1.pdf]

APPENDIX A – SEARCH STRATEGY

Database	Platform	Search Strategy	Date Run	# of results	Output
CINAHL	EBSCO	(Assertive Community Treatment<br>OR<br>mental health outreach<br>OR<br>Assertive Community Outreach<br>OR<br>Flexible Assertive Community Treatment)<br><br>AND<br><br>(Adult OR Young adult OR Middle age)<br><br>AND<br><br>emergenc*<br><br>DATE RANGE: 2014-2023	3/10/23	(33)	RIS<br>ENDNOTE
SCHOLAR	GOOGLE	Emergency department "assertive community treatment" "mental illness" "adult" "middle age" "young adult"	3/10/23	17	ENW<br>ENDNOTE
PSYCHINFO	OVID	Emergenc*.af.<br><br>AND<br><br>(Adult and young adult and Middle age).af.<br><br>AND<br><br>(Assertive Community Treatment or Flexible Assertive Community Treatment or Assertive Community Outreach or Mental Health Outreach).af.<br><br>DATE RANGE 2013 - 2023	3/10/23	6	OVID.CGI
PUB MED	National Library	"Assertive Community Treatment"<br><br>OR<br><br>"mental health outreach"	5/10/23	19	

		OR<br><br>“Assertive Community Outreach”<br><br>OR<br><br>“Flexible Assertive Community Treatment”<br><br>AND<br><br>Adult OR Young adult OR Middle age<br><br>AND<br><br>emergenc*<br><br>DATE RANGE 2014 – 2023			
MEDLINE		Assertive Community Treatment<br>OR<br>mental health outreach<br>OR<br>Assertive Community Outreach<br>OR<br>Flexible Assertive Community Treatment<br><br>AND<br><br>Adult OR Young adult OR Middle age<br><br>AND<br><br>emergenc*<br><br>DATE RANGE 2014 – 2023	5/10/23	33	

		No subject equivalents			
SCOPUS		("Assertive Community Treatment" OR "mental health outreach" OR "Assertive Community Outreach" OR "Flexible Assertive Community Treatment" AND "Adult" OR "Young adult" OR "Middle age" AND "emergency department" AND "mental illness" AND NOT prison AND NOT homeless AND PUBYEAR > 2013 AND PUBYEAR < 2024) AND (assertive AND community AND treatment) AND (EXCLUDE (SUBJAREA , "ECON") OR EXCLUDE (SUBJAREA , "AGRI") OR EXCLUDE (SUBJAREA , "BIOC") OR EXCLUDE (SUBJAREA , "BUSI") OR EXCLUDE (SUBJAREA , "DECI") OR EXCLUDE (SUBJAREA , "COMP") OR EXCLUDE (SUBJAREA , "ARTS") OR EXCLUDE (SUBJAREA , "ENVI"))	5/10/23	8	
Social Care online	SCIE online	ASSERTIVE COMMUNITY TREATMENT<br><br>ADULTS<br><br>EMERGEN*	29/10/23	0	
Cochrane		1 Cochrane Review matching Assertive Community Treatment in Title Abstract Keyword AND emergen* in Title Abstract Keyword - (Word variations have been searched)	29/10/23	1 (discarded wrong intervention)	
Social Work Abstracts	Proquest	noft("Assertive Community Treatment") (noft(emergency))<br><br>Date range limited 31/12/2013 - 01/01/2014<br><br>Peer reviewed only	29/10/23	57 (55 duplicates. 3 included)	
